# Metal-Organic Frameworks for the Development of Biosensors: A Current Overview

**DOI:** 10.3390/bios8040092

**Published:** 2018-10-16

**Authors:** Sergio Carrasco

**Affiliations:** Department of Organic Chemistry, Stockholm University, SE-106 91 Stockholm, Sweden; sergio.carrasco@su.se; Tel.: +46-08-162479

**Keywords:** metal-organic frameworks, polymer-based biosensors, MOF-based fluorescence biosensors, MOF-based electrochemical biosensors, MOF micro/nanostructuring

## Abstract

This review focuses on the fabrication of biosensors using metal-organic frameworks (MOFs) as recognition and/or transducer elements. A brief introduction discussing the importance of the development of new biosensor schemes is presented, describing these coordination polymers, their properties, applications, and the main advantages and drawbacks for the final goal. The increasing number of publications regarding the characteristics of these materials and the new micro- and nanofabrication techniques allowing the preparation of more accurate, robust, and sensitive biosensors are also discussed. This work aims to offer a new perspective from the point of view of materials science compared to other reviews focusing on the transduction mechanism or the nature of the analyte. A few examples are discussed depending on the starting materials, the integration of the MOF as a part of the biosensor and, in a deep detail, the fabrication procedure.

## 1. Introduction

In recent decades, the scientific community has focus its attention on rapid, sensitive, and selective analysis methods not only for qualitative but also for the quantitative determination of specific target molecules.

Nowadays, interest in the detection and quantification of several low-molecular weight organic compounds, as well as (bio)macromolecules, widely applied in daily life, has increased as some of them have demonstrated harmful effects on human health and the environment [1]. These compounds include food additives, drugs used in clinical and veterinary medicines, sometimes misleadingly, and even waste or by-products related to human and industrial activities. The total amount of molecules showing toxic, harmful, or carcinogenic properties and, in general, with negative impact on the health of living organisms, has dramatically grown in recent years. Thus, competent authorities work hard on the development of new severe legislations to minimize the impact of these compounds at different levels [2,3]. In that sense, the use of chemical biosensors for real-time detection of these analytes in different kind of samples ensures that the laws are obeyed, and the legislation objectives are fulfilled [4].

Different biosensor schemes have been developed based on a broad variety of organic and inorganic materials, such as silica nanoparticles [5] or metal colloids [6] and, more specifically, using highly engineered materials like molecularly imprinted polymers (MIPs) [7]; metal organic frameworks (MOFs) [8]; quantum dots (QDs) [9]; carbon derivatives such as fullerenes [10], graphene/graphene oxide [11], or nanotubes [12]; or even combinations of these supports to obtain hybrid materials with synergetic properties in order to overcome the limitations found using classical biosensors [13]. The final goal requires improving the limits of detection compared to previous conventional analyses and ensuring rapid and direct results, always looking for their recyclability and a stable signal performance with the minimum treatment of the sample and avoiding potential matrix effects.

## 2. Metal-Organic Frameworks

**Figure 1:**
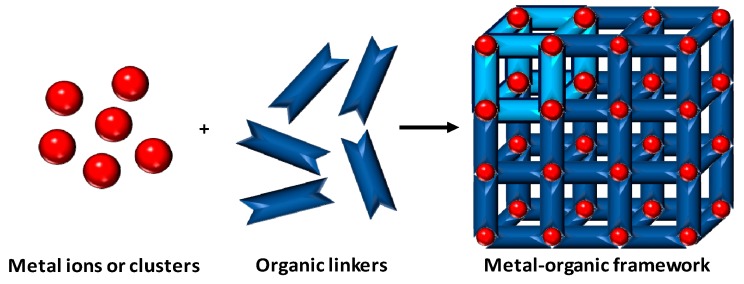
Scheme for the preparation of a MOF. Different metal ions or clusters are mixed together with organic linkers using a convenient solvent. Coordination polymerization takes place between the precursors, resulting in a cross-linked network showing potential voids.

Metal-organic frameworks are synthetic polymeric hybrid materials comprised of metal ions or metal clusters and an organic linker that assembles the structure together, somehow resembling zeolites in terms of surface area and crystallinity (Figure 1). Similar to porous coordination polymers, but showing crystalline structures, these materials are characterized by the presence of potential voids according to the IUPAC definition [14]. Besides the desirable porosity, MOFs can be defined as tailor-made materials, showing high compatibility with both organic and aqueous medias and can be considered as low-cost materials depending on the metal source employed for their preparation [15]. These coordination polymers can be characterized using a broad amount of techniques such as nitrogen adsorption porosimetry, electronic, optic and atomic force microscopies, powder X-ray diffraction, solid-state NMR, UV–vis and IR spectroscopies, among others [16,17]. Furthermore, the preparation of MOFs consists, in general, of conventional solvothermal syntheses that can be achieved employing autoclaves, with the possibility of the fabrication under microwave-assisted conditions to accelerate the kinetics of the coordination polymerization, increasing the overall yield, or even inside glass vials by mixing the precursors, depending on the fabrication requirements [18]. Moreover, in the recent years a new polymerization approach has emerged as a powerful fabrication technique to fine tune the size and morphology of the polymeric material based on its electrosynthesis onto the surface of conductive materials [19,20].

MOFs have been widely applied in different fields depending on the properties shown, such as catalysis [21], separation [22], purification [23], drug storage/delivery [24], gas storage [25], energy [26], and sensors development. In the further application, MOF materials can be considered typically as the recognition element of the sensor, while the selectivity can be given by different factors:

First, one should highlight the broad literature that can be found regarding the quantification of small organic molecules, even gases or atomic species. These first examples describe the use of MOFs as recognition elements in biological or chemical sensors based on their exclusion size discrimination capacity that depends on the porosity of the material and, in turn, on the linkers and metal precursors used for their fabrication [27]. On the other hand, the selectivity can arise from the interaction of the analyte with the linker or the structural metal. In the former approach, different forces can be considered, such as hydrogen bonding, hydrophobic (π-π) interactions, electron donor/acceptor interactions, or the formation of a dative covalent bonding [28,29]. When considering the metal-based recognition, it is mandatory that the node shows an open site where the target molecule can interact either via reversible bonds or electrostatically. Although MOFs can accomplish the role of recognition elements for the development of physical sensors, for example to measure temperature or moisture, regarding their capacity to adsorb water molecules, this is far from the scope of this review, and the reader is referred to the literature for further information [30]. However, it is noteworthy to mention that these are only a few examples compared to chemical sensors, where despite the parameter being measured is a physical magnitude of the media, the transduction of the signal can be triggered in a (opto/electro)chemical way [31].

The use of MOFs as recognition elements can be compared with other interesting polymeric materials, such as MIPs, with the exception of the inherent selectivity of the material, as a template molecule is used for the preparation of the latter polymer, and the polymerization mechanism, that follows a coordination or radical pathway, respectively. This comparison becomes interesting for the development of chemical biosensors, as MIPs, firstly described in 1949 [32], can be considered as mature materials respect to MOFs [33], but with remarkable similarities. Both polymeric materials show several advantages versus biomolecules as recognition elements [34]: higher stability, the possibility to get tailor-made materials, their compatibility in different harsh environments (organic, aqueous, high temperatures, extreme pH conditions, etc.) and with a fabrication cost lower than bioreceptors. However, some important drawbacks should be mentioned, like the slow binding kinetics offered by the synthetic materials, the analysis of targets without intrinsic measurable properties, the coupling of the MOF to the transducer element, and the possibility to perform multiplexed analyses. Also, the compatibility and stability should be considered, particularly when biomolecules are monitored as target analytes [35].

Sometimes, the lack of selectivity is the strongest issue that scientific community working on the development of new MOFs has to confront of, not only for sensing but for catalytic purposes [36]. The vast majority of the examples described in the literature concerning MOFs for (bio)sensing applications considers the material itself both as a receptor, regarding its ability to discriminate between different chemical species, and as a transducer, according to their capacity to experiment changes that can be measured to quantify the target molecule depending on its concentration. Several transduction schemes have been applied following, for example, optical, mechanical, or electrochemical mechanisms [37,38,39,40,41]. However, the vast majority of them are based on optical changes due to the insulating nature of MOFs [42]. For the reasons commented above, it is not surprising that the last efforts have been focused on the development of hybrid materials not only to enhance the selectivity of the materials and the transduction mechanism in order to fabricate more robust and reusable biosensors, but also for their use in further applications far from the scope of this review [43,44,45].

Despite the use of MOFs as raw materials for biosensor development still represents a broad literature production, in recent years the remarkable issues found have resulted in deep investigations to combine their properties with other materials. Among these disadvantages, one should highlight the control of the size, shape and morphology in a reproducible way. Their preparation along with other organic and inorganic materials have considerably improved the performance of MOFs for biosensor development [46].

## 3. State of the Art

**Figure 2:**
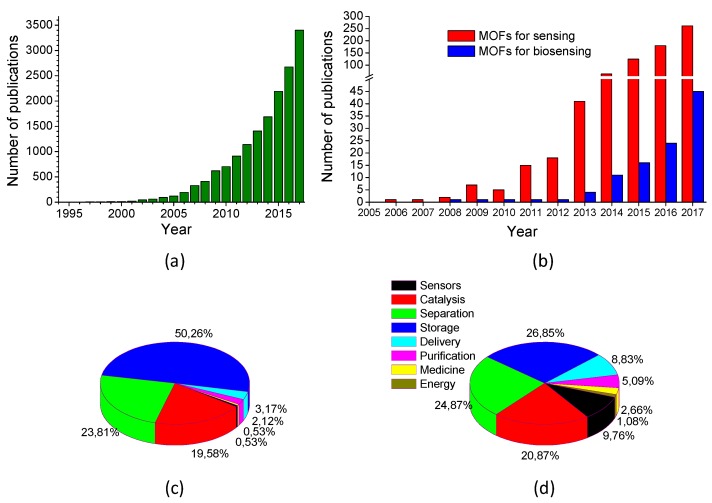
Number of publications of: (**a**) MOFs in the period 1994–2017; (**b**) MOFs used for chemical sensor or biosensor development in the period 2005–2017. Distribution of MOF publications in different fields for the years: (**c**) 2007; and (**d**) 2017. Source: Web of Science.

Publications in the MOF field have increased exponentially since Yahgi et al. presented the first work concerning these coordination polymers in 1994 (Figure 2a) [33]. However, the first sensor-related work involving MOFs was published in 2002 [47], and the first example for biosensing can be found in 2008 (Figure 2b) [48]. Comparing the literature production between different applications of MOFs in 2007 (Figure 2c) and 2017 (Figure 2d), the percentage of MOF-based sensor works represented 0.5% and 9.8%, respectively, indicating the growth interest in this field. Particularly, for the last year biosensors significantly represented the 17.2% of the overall MOF-based sensor production. This value can be compared with the 5.6% in 2012 that, under the point of view of this author, is the result of the maturing of MOF technology and its combination with the novel micro-/nanostructuration techniques for stablishing novel robust and reproducible methods for the quantification of high-impact biomolecules in real samples. A few words regarding other fields should be mentioned, such as the constant production of MOFs for catalysis and separation that show a stagnant growth during the last decade, or the significant increase on sensor, purification, drug delivery, medicine, or even energy-harnessing and energy-storage fields to the detriment of storage applications.

The scope of this review is the use of metal-organic frameworks for the fabrication and development of such biosensor methodologies described above. Despite the large amount of reviews that one can found in the literature, the perspective of this review is focused on the material itself, its characteristics and how the MOF is structured in order to achieve the final goal for biosensing purposes, with special emphasis on the publications found in the literature in 2017 and 2018, as can be considered as the most promising works that may pave the way for the new generation of biosensors, according to the literature searching performed. Other previous reviews have been published following different approaches and classifications, based on the transduction mechanism [36,49], the preparation procedure [50,51], biomolecule immobilization procedure [52] and, more extensively, on the nature of the analyte [53,54,55,56,57,58].

## 4. MOFs for Biosensing

As stated before, in this section MOF-based biosensor publications are divided in three main different groups according to their complexity and regarding the fabrication techniques for sensor development. These groups concern mainly to those polymers prepared following conventional syntheses and applied as synthesized, known as raw MOFs; the same polymers prepared following ‘grafting to’ approaches, i.e., by physical or covalent attachment to a specific substrate that acts as the transducer or improves the chemical signal; and those materials prepared using other substrates as ‘seeds’ or cores in a ‘grafting from’ approach.

### 4.1. Raw MOFs

This section includes all these MOF-based biosensor schemes where the material is used after its synthesis without further modifications, and it is prepared in the absence of other potential supports that can be used as cores for the crystal growing. Table 1 summarizes some of these materials. As expected, due to the poor conductive properties of MOFs, transduction mechanisms are mainly based on optical signals.

The preparation of fluorescent Zn-MOFs resulted in the development of an optical biosensor for the detection of pesticides [59]. The material was fabricated using Zn(NO_3_)_2_ and 1,2,4,5-tetrakis(4-carboxyphenyl) benzene (H_4_TCPB) as linker. The fluorescence quenching was measured upon parathion-methyl adsorption and the biosensor showed a limit of detection (LOD) of 0.12 μg/kg and a linear range of 1 μg/kg–10 mg/kg for this analyte, with low cross-reactivity to other nitroaromatics, and finally applied to the analysis of spiked lake water with high recoveries (> 93%) and low relative standard deviation (RSD, 5.9%).

Valekar and colleagues presented a MOF with peroxidase-like activity used for the fluorescence detection of choline and acetylcholine [60]. The hydrothermal synthesis of MIL-100(Fe) consisted on the mixture of metallic Fe, trimesic acid (BTC), HF, and nitric acid in water. MOF was amine grafted on the coordinatively unsaturated sites using different diamines. The biosensor is based on acetylcholine esterase and choline oxidase that generate H_2_O_2_ after their catalytic oxidation from acetylcholine. This activated the amino-functionalized MOF to convert the substrate Amplex UltraRed (AUR) into a highly fluorogenic probe that was monitored in a microplate reader. The analytes choline and acetylcholine were detected down to 0.027 μM and 0.036 μM, and both linear ranges were very limited, being 0.5–10 and 0.1–10 μM, respectively. The biosensor exhibited a strong pH- and temperature-dependent behavior, but it was successfully applied to the quantification of these analytes in milk and serum samples with excellent recoveries >97%. No further investigations on the recyclability or cross-reactivity were performed for this biosensor.

A novel Cu-MOF showing large void space was prepared from CuSO_4_, 1,2-bis(4-pyridyl)ethylene (bpe) and a tailored tricarboxylic linker *N*-(3,5-dicarboxylbenzyl)-(3-carboxyl) pyridinium bromide (H_3_DcbcpBr), in water [61]. Due to the presence of benzene rings, free carboxylates, Cu(II) cation centers, and positively charged pyridinium within the polymeric matrix, the authors suggested the potential interaction of this MOF with negatively charged nucleic acids. They assayed the interaction between the MOF and a carboxyfluorescein (FAM)-labeled or 5(6)-carboxyrhodamine, triethylammonium salt (ROX)-tagged single-stranded DNAs (ssDNAs), showing a complementary sequence for Dengue and Zika viruses RNAs, respectively. By measuring the fluorescence quenching efficiency of the MOF, the LOD values found were 332 ppm and 192 ppm for Dengue and Zika viruses, with linear ranges 1–60 nM and 0.5–70 nM, respectively. The multiplexing detection of both viruses was possible using synchronous scanning fluorescence spectrometry, as FAM and ROX signals can be measured simultaneously and the biosensor showed that LODs were slightly improved to 184 ppm and 121 pM. The observed cross-reactivity was negligible when using other fluoresce-labeled DNA sequences, but neither reproducibility between different batches nor recyclability studies were performed in this work. A similar biosensor scheme was proposed by Qiu and colleagues for the detection of five different gastric cancer associated micro RNAs (miRNAs) [62]. For this purpose, a 1D Cu-MOF was prepared using Cu(NO_3_)_2_ and a sodium salt of 1-(3,5-dicarboxybenzyl)-4,4′-bipyridinium bromide (H_2_dcbbBr) in water following a solvothermal synthesis. LODs obtained were in the range of 91–559 pM with negligible cross-reactivities towards to four potential interfering miRNA sequences.

### 4.2. “Grafting to” Approaches

Within this section, the reader will find different fabrication procedures widely used for the development of biosensors regarding how the MOF participates in the recognition or transduction mechanism, but following the same “grafting to” approach, i.e., the immobilization of the polymeric material onto the surface of different supports. The distribution herein has been performed according to the complexity of the material. Section 4.2.1 inquiries about simple physical or chemical deposition of similar MOFs than those described as raw materials in the previous section, in general, for electrochemical purposes. The substrate, usually an electrode, confers to the system the electronic conductivity required for the development of this kind of biosensors. The vast majority of biosensors published to date belong to this group. Section 4.2.2 differs from the previous regarding the final morphology of the material. While the former group includes all these MOFs obtained in bulk and non-controlled size, the latter gathers 2D-nanosheet morphology that facilitates the integration and their grafting to other bidimensional supports. Section 4.2.3 includes all of these bulk MOFs that, after their synthesis, are impregnated with different metal salts and further reduced to obtain metal nanoparticles within the matrix voids. Finally, Section 4.2.4 summarizes a few examples where bulk MOFs are previously calcined in order to improve, in general, their electric properties.

#### 4.2.1. Bulk MOFs

As stated before, this group represents the vast majority of MOF-based chemical sensors and biosensors described in the literature for the last decade. Although Table 2 shows different examples published in 2017 and 2018, hundreds of examples can be found in the literature during the last years.

**Figure 3:**
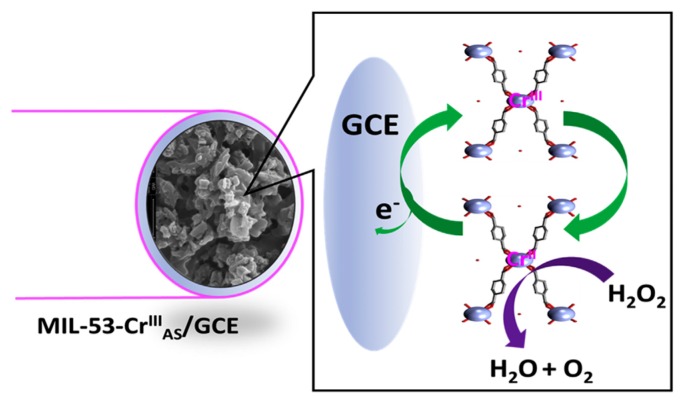
Proposed mechanism for the reduction of H_2_O_2_ at MIL-53(Cr(III))/GCE. Reprinted from reference [83]. Copyright 2018, with permission from Elsevier.

Lopa et al. presented an electrochemical biosensor for the quantification of H_2_O_2_ based on MIL-53(Cr) [83]. The MOF was prepared following a microwave-assisted solvothermal synthesis mixing CrCl_3_ and terephtalic acid (TPA) in water and further treated with NaOH. A suspension of this material in Nafion and ethanol was drop casted onto the surface of a glassy carbon electrode (GCE). The modified material showed electrocatalytic properties for the reduction of H_2_O_2_ in basic media, proposing that, firstly, Cr(III) species are reduced to Cr(II), and MOF-53(Cr(II)) is the responsible for the analyte reduction, regenerating the MOF-53(Cr(III)) (Figure 3). The biosensor was stable after more than 200 cycles without cross-reactivity and showed a detection limit of 3.52 μM and a short linear range of 25–500 μM. Spiked serum samples were analyzed with recoveries in the range 98.33–101.62%.

By the combination of a bulk MOF and metal nanoparticles, miRNA-122 was successfully quantified in serum samples using an electrochemical transduction scheme [84]. For that purpose, authors prepared the polymer MIL-88(Fe)-NH_2_ in a solvothermal synthesis using FeCl_3_ and 2-aminoterephtalic acid (2ATPA) in DMF and acetic acid (AcOH). In this case the metal nanoparticles (PdNPs) were not created in situ but prepared in a separated step and anchored to the polymeric matrix afterwards via the amino groups on the surface. Streptavidin was attached to the surface of the nanocomposites and finally incubated with the biotinylated probes to obtain the tracer label able to mimic the peroxidase activity and thus assisting the electrochemical signal from the 3,3′,5,5′-Tetramethylbenzidine (TMB) (peroxidase substrate)-H_2_O_2_ catalysis reaction. On the other hand, a GCE electrode was functionalized with AuNPs@N-G (nitrogen-doped graphene sheets) and the capture probe was attached by self-assembly to AuNPs. Finally, the modified electrode is incubated with solutions containing the analyte and, by last, with the tracer bioconjugates. The biosensor achieved a LOD of 0.003 fM and a linear range of 0.01 fM–10 pM and was applied to both spiked serum and human blood samples, although neither stability nor cross-reactivity studies were performed.

Using the same MOF as described before, He et al. proposed an electrochemical biosensor with amplified detection for the ion Pb^2+^ [85]. MIL-88(Fe)-NH_2_ was incubated with H_2_PtCl_6_ and Na_2_PdCl_4_ and the metal salts were rapidly reduced with NaBH_4_. It is expected that this quick reduction procedure yielded PtPdNPs attached to the surface of the polymer but not within the polymer pores.

The material was functionalized with the mercapto group labeled hairpin DNA that can match with the newly generated single-strand DNA, used as signal tag. On the other hand, the GCE electrode was modified with a reduced graphene oxide (GO)-AuNPs composite used as biosensor platform for immobilizing streptavidin and lately the biotin modified substrate strand. The following step consisted of the incubation of the electrode with the catalytic strand to create the Pb^2+^-specific DNAzyme. In the presence of the ion, the system was activated and the substrate strand cleaved. Amplification was achieved after adding MOF-bioconjugates allowing a LOD of 2 pM and a linear range of 0.005–1000 nM. Other metal ions did not show cross reactivity even at a concentration which was 100-fold of the Pb^2+^ concentration. The biosensor was applied to different water samples (spiked, reservoir, well, and tap) showing recoveries >96.00% and RSD < 0.27%. However, the main limitation of the biosensor may be the large incubation times required to obtain the electrochemical signal that included, at least, two mandatory steps: incubation with the analyte (45 min) and amplification of the signal (2 h).

Yu and colleges reported a Hg(II)-triggered electrochemiluminescence (ECL) biosensor based on a Ru-MOF for the detection of miRNA-155 [86]. The solvothermal synthesis of the material was performed using Zn(NO_3_)_2_, 4,4′-Biphenyldicarboxylate (BPDC), adenine and Ru(bpy)_3_Cl_2_ in a mixture of HNO_3_, DMF, and water. MOF was functionalized in the following step using (3-aminopropyl)-triethoxysilane (APTES) to introduce an amino moiety within the polymeric matrix. To obtain the Ru-MOF conjugates, the previous particles were incubated with a carboxyl-modified oligonucleotide (I). In parallel, a GCE electrode was incubated with the salt HAuCl_4_ and electrochemically reduced to obtain a gold film. A thiol-modified oligonucleotide (H1) was deposited onto the modified electrode. The next step consisted on the incubation of the biosensor with a second oligonucleotide (H2) and miRNA-155 and, finally, the addition of I-RuMOF-conjugates. The integrity of the MOF is strongly damaged in a selective way using Hg(II) ions, releasing Ru(II)(bpy)_3_ ions responsible for the ECL signal. A low LOD of 0.3 fM was achieved with a linear range of 0.8 fM–1 nM. The biosensor was used for the quantification of miRNA-155 in spiked serum samples with recoveries >97.17% and RSD < 4.12%. The stability was monitored after 10 consecutive cycles, but no further studies were performed. One could expect that the sensing material cannot be regenerated after the addition of larger amounts of Hg^2+^ and more cycles, that may result in lower sensing life-times compared to other biosensors. Another limitation that should be highlighted is the large incubation time required to get the ECL signal, that raise each measurement up to 4 h.

A high-advanced potential point of care biosensor based on fluorescence quenching was proposed by Huang et al. for the quantification of different cancer biomarkers, gene p53 and prostate specific antigen (PSA) [87]. A PCN (porous coordination network) MOF-type was thermally prepared using ZrCl_4_, tetrakis (4-carboxyphenyl) porphyrin (TCPP) and benzoic acid (BA) as modulator in DMF. AuNPs were grown over the surface of the needle-shaped MOF crystals and, in the last step, an ultrathin graphene oxide (GO) layer was immobilized over the hybrid material in the presence of hydrazine. A fluorescent-labeled ssDNA probe was synthesized for the recognition of p53 gene while a dye-labeled aptamer was prepared for que quantification of PSA. The strategy for sensing consisted of the adsorption of the dye-labeled ssDNA onto the material previously prepared. In the presence of the analyte, a specific hybridization occurs between the target DNA and the fluorescent ssDNA, resulting in the formation of double-stranded DNA (dsDNA). Due to its lower affinity for the material than that of ssDNA, the former is detached from the surface triggering fluorescence recovery of dye molecules. The gene was detected with a detection limit of 0.005 nM and a linear range of 0.01–10 nM in 10 min and the LOD value for PSA was 0.01 ng/mL with a linear range of 0.05–10 ng/mL after 35 min, being both of them analyzed in spiked serum samples.

#### 4.2.2. Nanosheets

Some modifications in the preparation procedure of bulk materials allow to obtain bidimensional MOF layers showing improved integration properties onto the surface of different substrates. Table 3 summarizes some examples concerning these MOF-based biosensors.

Shao et al. proposed an ECL biosensor for the detection of miRNA-141 [102]. The system consisted of the preparation of a capture unit based on magnetic core–shell nanoparticles covered with a silica layer and decorated with gold nanoparticles, functionalized with capture DNA (cDNA) in a further step. The capture unit was attached to the surface of a GCE electrode. In parallel, the signal unit based on Ru-MOF nanosheets was built up using the complex [Ru(dcbpy)_3_]^2+^ and Zn(NO_3_)_2_ in a mixture of propanol:water and capped with signal DNA (sDNA). After the recognition of the miRNA-141 by the capture unit, the system was developed using the signal unit, yielding a limit of detection of 0.3 fM and a wide dynamic range (DR) of 1 fM–10 pM, being successfully applied to spiked human serum samples with recoveries up to 110%.

An interesting optical Förster resonance energy transfer (FRET)-based aptasensor for the quantification of chloramphenicol (CAP) was developed by Yang et al. [103]. Cu-MOF nanosheets were prepared using Cu(NO_3_)_2_ as metal source and TCPP as ligand, in the presence of trifluoroacetic acid (TFA) and polyvinylpyrrolidone (PVP) in a mixture DMF:ethanol. The recognition was based on circular strand-replacement DNA polymerization using the Cu-MOF as quencher and a designed aptamer hairpin probe whose stem is open while binding the target. Once it is open, it binds the primer and polymerase generates dsDNA, that can be bound to SYBR green I to generate the optical signal. The biosensor showed a linear response range of 0.001–10 ng/mL, with a LOD of 0.3 pg/mL. It was applied for milk and fish samples and exhibited a high selectivity compared to other antibiotics.

**Figure 4:**
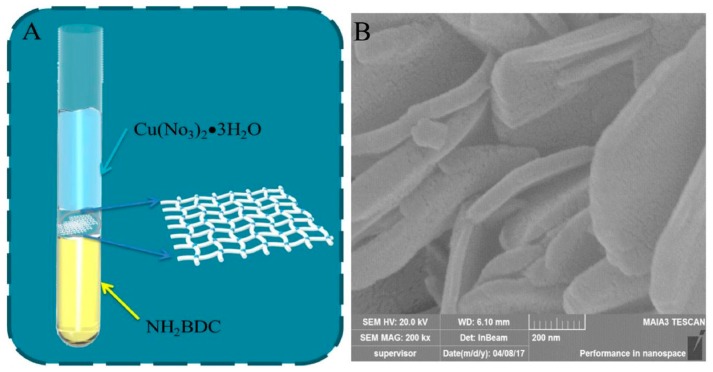
(**A**) Schematic diagram of the bottom-up strategy for the synthesis of Cu-MOF nanosheet; (**B**) SEM image of the material. Reprinted from reference [104], Copyright 2018, with permission from Elsevier.

Following a bottom-up synthesis strategy, amino-functionalized Cu-MOF nanosheets were fabricated for detection of hypoxanthine (HXA) by fluorescence quenching [104]. The sensing material was fabricated under static conditions, mixing carefully different solutions containing the precursors. The first solution consisted of 2ATPA in DMF:CH_3_CN, over which a mixture of the same solvents was slowly added to separate the linker from the metal precursor, Cu(NO_3_)_2_, dissolved in the third layer (Figure 4). These MOF nanosheets exhibited peroxidase mimic properties and were incubated in a solution containing the analyte and xanthine oxidase. After the addition of *o*-phenylenediamine the fluorescence of the material was measured. The main drawback of the proposed biosensor is the incubation time required to obtain the signal (55 min) but showed a wide linear range of 10–2000 μM and a LOD of 3.93 μM, being successfully applied to the quantification of hypoxanthine in fish samples, with negligible cross-reactivity to potential interferences.

#### 4.2.3. Metal Nanoparticles @ MOF

The in situ reduction of metal salts within the polymeric matrices yields bulk materials showing metal nanoparticles embedded that, after their grafting to the substrate, results in lower LOD MOF-based biosensors compared to the corresponding bulk-based materials, particularly increasing the conductivity of the polymer for the development of electrochemical biosensors. Some of these examples are presented in Table 4.

Wang and colleagues proposed a new electrochemical biosensor for glucose quantification using Ag@ZIF-67 (zeolitic imidazolate framework) nanocomposite [107]. First, the MOF was synthesized at room temperature mixing Co(NO_3_)_2_ and 2-methylimidazole (2MI) in a mixture of alcohols. The polymer was thermally treated to evacuate the internal pores and then incubated with AgNO_3_ and further reduced with NaBH_4_ to obtain AgNPs. A polished GCE electrode was covered by a suspension of this material in Nafion. Glucose oxidation was monitored in basic media and it was demonstrated that the presence of silver nanoparticles enhanced both the conductivity and catalytic activity. The biosensor was able to detect the analyte down to 0.66 μM with a linear range 2–1000 μM. Potential interfering species like uric or ascorbic acid did not affect the glucose recognition at their conventional biological levels. The biosensor was stable after one-month of storage or 25 measuring cycles.

An electrochemical sandwich-type immunosensor for the detection of the biomarker galectin-3 (Gal-3) was performed using Au@MIL-88(Fe)-NH_2_ functionalized GCE electrodes [108]. The synthesis of the MOF was performed mixing FeCl_3_ and 2ATPA in DMF and AcOH. The material was incubated with HAuCl_4_ and then reduced with NaBH_4_ to create the AuNPs@MOF particles. In the last step, they were combined with nitrogen-doped graphene nanoribbons (N-GNRs), that have demonstrated to improve the conductivity performance of surface functionalized electrodes, to obtain the final hybrid material. The surface of the functionalized electrode was loaded with Gal-3-(antibody)Ab_2_ and incubated with the analyte. On the other hand, methylene blue crystals decorated with AuPtNPs, and further functionalized with Gal-3-Ab_2_ were prepared in order to perform the sandwich-type assay and get the electrochemical signal. The linear response was found to be 100 fg/mL–50 ng/mL, with a LOD of 33.33 fg/mL. Potential interfering species produced no more than a 1.55% of signal variation at high concentration levels and the biosensor showed a good reproducibility (RSD < 2.75%) between different electrodes. Recoveries higher than 97.99% were achieved when analyzing spiked serum samples.

Considering the plasmonic properties of silver nanoparticles, Zhang et al. proposed not only an electrochemical biosensor but also a surface plasmon resonance (SPR)-based biosensor for the selective quantification of carcinoembryonic antigen (CEA) [109]. UiO-66 (Universitetet i Oslo) was prepared using ZrCl_4_ and TPA in DMF. After the addition of TFA and HCl as modulators, reaction took place at 120 °C for 24 h. The polymer was incubated with AgNO_3_ and CEA-aptamer, and further reduced with NaBH_4_. A suspension of this material was prepared and it was drop casted onto an Au electrode and a SPR chip. The addition of CEA-aptamer conferred the selectivity to the material as it specifically binds to the analyte after the immobilization of the aptamer strands within the nanocomposite. Electrochemical biosensor was able to measure in a linear range of 0.01–10 ng/mL with a LOD of 0.56 pM in electrochemical impedance spectroscopy (EIS) mode and 0.31 pM in differential pulse voltammetry (DPV) mode, while SPR biosensor yielded a linear range of 4.0–250 ng/mL and a LOD of 0.3 ng/mL. However, the signal in the former biosensor was obtained immediately while in the SPR biosensor it is required to equilibrate the baseline signal consuming 6 h of the assay. Electrochemical biosensor showed negligible cross-reactivity to other potential interferents, even at 100-fold concentration of CEA, it was stable after nine days and the reproducibility between five different batches was high, with RSD < 2.75%. Moreover, chips can be regenerated up to seven cycles with an easy alkaline treatment. Finally, spiked serum samples were analyzed with recoveries higher than 97.6%.

**Figure 5:**
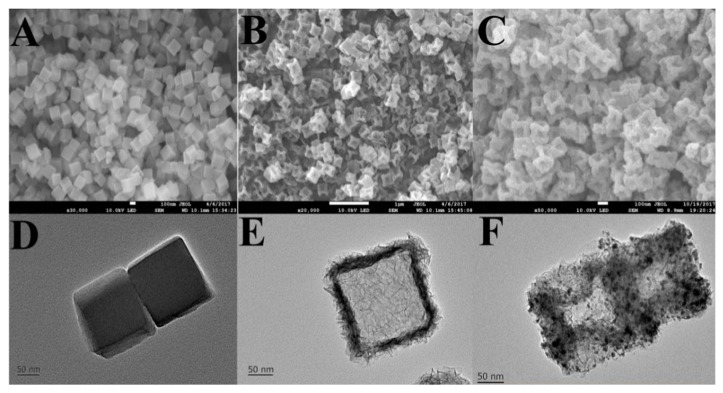
Scanning electron micrographs (SEM) of: (**A**) ZIF-67 nanocubes; (**B**) hollow nanoboxes; (**C**): AuPt-decorated nanoboxes. Transmission electron micrographs (TEM) of: (**D**) ZIF-67 nanocubes; (**E**) hollow nanoboxes; (**F**): AuPt-decorated nanoboxes. Reprinted from reference [110], Copyright 2018, with permission from Elsevier.

A hollow nanobox-MOF/AuPt alloy is used for the development of an electrochemical immunosensor for the quantification of the protein LAG-3 [110]. In the first step, ZIF-67 nanocubes were prepared in water using Co(NO_3_)_2_ and 2MI in the presence of cetyltrimethyl ammonium bromide (CTAB) as surfactant (Figure 5A,D). Hollow nanoboxes were created upon the addition of Ni(NO_3_)_2_ to a solution containing the previous MOF and ultrasonic/thermal treatment (Figure 5B,E). The AuPt alloy layer was created using H_2_PtCl_6_ and HAuCl_4_ in the presence of the previous hollow nanoboxes and NaBH_4_ as reducing agent (Figure 5C,F). This material was immobilized onto the surface of rGO-SnO_2_ nanosheets and further deposited on a modified GCE electrode. Streptavidin selectively binds the alloy and the electrode was then incubated with biotin-modified antibodies. Finally, the sandwich-type immunoassay was performed by incubating the modified electrode with LAG-3 and antibody-modified silica nanoparticles to enhance the sensitivity of the biosensor, amplifying the electrochemical signals by the reduction of H_2_O_2_. The biosensor was applied for the determination of the analyte in serum samples, showing a lack of cross-reactivity to other proteins, with a LOD of 1.1 pg/mL and a DR of 0.01 ng/mL–1 μg/mL.

#### 4.2.4. Pyrolysis/Calcination

Considering electrochemical applications, the calcination of MOFs has demonstrated to be a very efficient procedure to improve the features of the sensing process as the conductivity of the hybrid material is considerably increased. Shu et al. prepared a Ni-MOF based on TPA and NiCl_2_ that was calcined to obtain the hybrid Ni-MOF/Ni/NiO [115]. AuNPs were mixed with the previous material and further deposited onto the surface of a GCE electrode. The nanoenzymatic biosensor was applied for the quantification of glucose in serum samples according to its electrocatalytic activity towards glucose oxidation in alkaline media with a LOD of 0.1 μM and a linear range of 0.4–900 μM. The measurement of other species resulted in little current responses and the biosensor was applied to the quantification of glucose in serum samples.

Three carbon composites were prepared after thermal annealing of different 2ATPA-based MOFs showing Fe^3+^, Zr^4+^, and La^3+^ as structural metals for the electrochemical detection of methyl parathion [116]. The analysis of the resulting materials revealed that the structure of the original MOFs was maintained and carbon supported-metal oxide nanocomposites were created. Particularly, the MOF fabricated with lanthanum resulted in a wool-ball-like structure that enhanced the electrochemical activity of the biosensors. Calcined MOF was mixed with Nafion and deposited onto the surface of a carbon paste electrode (CPE). Acetylcholinesterase was immobilized on the functionalized electrode. The biosensor principle was based on the measurement of the inhibition rate of the analyte in the presence of fixed amounts of acetylthiocholine chloride. DRs were 10^−12^–10^−8^ g/mL, 5 × 10^−13^–5 × 10^−9^ g/mL, and 10^−13^–5 × 10^−9^ g/mL, with LODs of 3.2 × 10^−13^ g/mL, 1.8 × 10^−13^ g/mL, and 5.8 × 10^−14^ g/mL using the Fe, Zr, and La-calcined MOF materials. Although the biosensor seemed to be stable after one month, the original signal decreased ca. 20% and cross-reactivity was not tested in this work.

The same strategy was also used by Haldorai et al. to fabricate an electrode for the selective sensing of glucose [117]. In this case, ZIF-67 was created from CoCl_2_ and 2MI in methanol. After carbonization, nanoporous carbon (NPC)-Co_3_O_4_ was obtained showing a high specific capacitance, good rate capability, and long-term charge/discharge cycling than other materials. This material was deposited onto a GCE electrode, although authors do not describe the procedure to get the modified electrodes (deposition/immobilization). The biosensor offered a limited linear range of 5 × 10^−12^–2.05 × 10^−10^ M compared to previously described biosensors for glucose based on different materials but the lowest LOD from all of them, 2 × 10^−12^ M. Neither potential interfering species nor other ions in a large excess produced significant changes in the electrochemical signal. A decrease of 3.5% regarding the original signal was found after 100 cycles and the biosensor was applied for the analysis of blood serum samples with recoveries higher than 98.5%. However, no reproducibility between different electrodes was evaluated.

Some other ZIF MOFs have been used for similar biosensor purposes after their calcination due to the interesting photoelectrochemical (PEC) properties observed in the carbon-based materials obtained. For example, Yang and colleagues published an interesting work using nitrogen-doped NPC-ZnO nanopolyhedra for the selective detection of alkaline phosphatase in spiked human serum samples with recoveries higher than 97.1% [118]. ZIF-8 was synthesized using Zn(NO_3_)_2_ and 2MI as precursors in methanol at room temperature. Carbonization occurred at 600 °C under inert atmosphere and the nanoparticles were further drop-casted onto the surface of an indium tin oxide (ITO) electrode. The biosensor was directly incubated in the presence of the analyte and in a second step with a fixed amount of an ascorbic acid salt in order to increase the signal as a consequence of the hydrolyzation of this salt by the analyte. Finally, the photocurrent was measured yielding a LOD of 1.7 U/L and a linear range of 2–1500 U/L. Although the authors optimized the time of this reaction in 20 min, they do not specify the total time required for the whole assay. Nevertheless, they evaluate the reproducibility with 6.3% RSD, the stability, up to two weeks for three independent measurements and the cross reactivity to other interfering proteins, measuring the analyte signal that was decreased ca. 10%.

Further examples can be found in Table 5.

### 4.3. “Grafting From” Approaches

Although the terminology ‘grafting from’ is widely used to refer the concept where a second material is grown onto the surface of a substrate to form, for example, bidimensional hybrid layers, or a discrete micro-/nanoparticle, in a core–shell fashion, and using materials of different nature, traditionally it is employed when polymeric materials are considered. This methodology enhances the mechanical properties of the final material compared to those obtained by “grafting to” approaches, as the integration of both core and shell is higher thus allowing the fine control of the thickness and morphology. In general, this approach is followed when synergetic or complementary properties are required and cannot be achieved by using only one of them separately. Either increase the conductivity or enhance the optical properties of the MOFs are the main aspects to be fulfilled in the development of a biosensor. This section is distributed according to the nature of the core/substrate, using both metal/metal oxides and carbon-based materials.

#### 4.3.1. Metal/Metal Oxide-Based Cores

Table 6 gathers the most important examples described for this kind of hybrid materials within the last years.

A colorimetric biosensor based on Fe_3_O_4_@MIL-88(Fe) was prepared by Zhang and colleagues to determine glutathione in serum samples [121]. For this purpose, mercaptoacetic acid-modified magnetic nanoparticles were mixed with the precursors, FeCl_3_ and TPA, in DMF and in the presence of NaOH to deprotonate the carboxylic acid moieties of the linker and enhance the interaction of the carboxylates with iron clusters. The material was suspended in a solution containing glutathione, H_2_O_2_ and methylene blue as indicator in a Fenton-like reaction. After removing the material with an external magnet, the UV–vis spectrum of the solution was acquired. Since the strict point of view of a biosensor definition, the material is not participating here neither as a receptor nor as transducer but this example highlights the importance of preparing core–shell materials to accelerate the overall process. The assay required 1 h according to the incubation steps and yielded a LOD of 36.9 nM with a DR of 0.55–3 μM.

Using also magnetic nanoparticles as cores, a highly engineered material was prepared for the fabrication of a fluorescence biosensor selective to ochratoxin A (OTA), with its successful application to quantify the mycotoxin in corn samples [122]. Fe_3_O_4_ nanoparticles were suspended in a mixture 1:1 (*v*/*v*) EtOH:H_2_O containing Cu(NO_3_)_2_, BTC and graphitic g-C_3_N_4_. After the solvothermal synthesis, the material was loaded with a FAM-labelled aptamer and further incubated with solutions containing different concentrations of the analyte. It is worth mentioning that the material itself participates only as a support for the controlled release of the aptamer, that shows higher affinity constants towards to the analyte than for the hybrid material. The high selectivity of the recognition element resulted in negligible cross-reactivity to other mycotoxins and the biosensor proposed yielded a LOD of 2.57 ng/mL with a DR of 5–160 ng/mL. The repeatability studies showed 2.5% RSD within six measurements and recoveries in real samples of >96.5%. However, as in the previous case, this is a typical example where the material is not acting neither as recognition element nor as transducer.

Based on the peroxidase-like activity of ZIF-8, a core–shell material using CaCO_3_ as template was obtained for the development of an electrochemical biosensor for glucose detection in serum samples [123]. In the first step, a shell based on polydopamine (PDA) was created onto the surface of the previous microparticles showing GO within the crystal structure. A second layer of the MOF material was prepared by mixing a suspension of this composite with Zn(NO_3_)_2_ and 2MI in a mixture EtOH:H_2_O. The hybrid material, showing magnetic properties, was mixed with a suspension of reduced rGO nanosheets and the core material was etched by acidic treatment, resulting in GO/PDA/ZIF-8@rGO hollow microcapsules. The final composite was deposited onto the surface of a GCE electrode and further used for glucose sensing. Glucose diffuses inside the microcapsules and is reduced by GO to produce H_2_O_2_, species that are in turn reduced by ZIF-8 as a consequence of its mimetic horseradish peroxidase activity where graphene nanosheets enhance the electron exchange between the MOF and the electrode. A DR of 1 μM–3.6 mM and LOD of 0.333 μM was obtained. None of the potential interferents investigated caused any notable response on the biosensor and the signal was stable after 25 consecutive cycles, with a reproducibility between different batches of 2.5% RSD. Moreover, after 15 days the response was 95% the initial signal and the recoveries in serum samples were >92.3%.

The application of QDs is also widely reported for the fabrication of luminescent biosensors based on their fluorescence quenching when used as cores. Wang et al. used this approach to elaborate a biosensor for the selective detection of H_2_O_2_ and, indirectly, for the quantification of urate and glucose oxidase [124]. A suspension of PVP-coated CdTe QDs in water was mixed with the MOF precursors, Zn(NO_3_)_2_ and 2MI. Due to the size-selective permeability shown by ZIF-8 towards to H_2_O_2_, both oxidases and substrate had very little effect on the fluorescence quenching of the QD. The hybrid material was mixed with urate or glucose oxidase and in the presence of uric acid or glucose, respectively, these molecules were reduced in an enzymatic reaction to produce H_2_O_2_, that was monitored with a DR of 1–100 nM and a LOD of 0.29 nM and recoveries larger than 97.2%. The biosensor also provided the possibility to quantify the amount of urate oxidase (DR: 0.1–50 U/L; LOD: 0.024 U/L) and glucose oxidase (DR: 1–100 U/L; LOD: 0.26 U/L) in serum samples. Different amino acids and ions that could be considered as potential interfering species were tested without a significant change on the fluorescence of the material, showing a good repeatability after seven measurement days. However, the reproducibility between different batches was not tested in this work.

**Figure 6:**
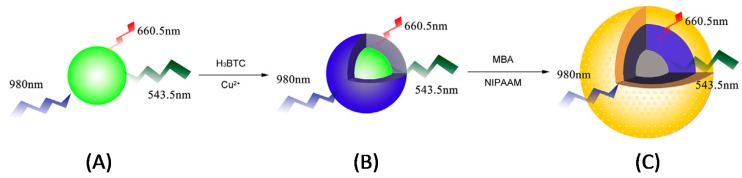
Fabrication scheme of: (**A**) Y,Yb,Er-UCNPs; (**B**) Y,Yb,Er-UCNPs@MOF; (**C**) Y,Yb,Er-UCNPs@MOF@MIP. Reprinted from reference [125], Copyright 2016, with permission from Elsevier.

A ternary up-conversion nanoparticles-based UCNPs@MOF@MIP hybrid material was prepared by Guo and colleagues for the development of a fluorescence-based biosensor selective to bovine hemoglobin (BHB) [125]. UCNPs were fabricated using Y, Yb, and Er salts and showed emission at 543.5 nm when exciting them at 980 nm, using the former wavelength for quantification purposes (Figure 6A). These nanoparticles were covered with polyacrylic acid (PAA) and used as the signal reporter. In a further step, a thin MOF layer of HKUST-1 (Hong Kong University of Science and Technology) was grown upon the addition of BTC and Cu(NO_3_)_2_ to a suspension of UCNPs@PAA in DMF:EtOH (Figure 6B). Over the hybrid QDs@MOF nanoparticles a new MIP layer based on *N*,*N*-methylenebisacrylamide (MBA) and *N*-isopropyl acrylamide (NIPAAM) was fabricated in the presence of the target analyte in order to confer the selectivity to the biosensor (Figure 6C). The first monomer was used for the creation of rigid pockets around the template molecule while the latter was used as functional monomer to induce the formation of selective H-bond interactions during the rebinding step, as well as to confer thermosensitive properties to the final material. Herein, MOF played the role of the spectator between the sensing material itself, UCNPs as transducer elements, and the recognition element, MIP layer, enhancing the mass transfer properties of the hybrid compared to those MIP materials prepared in bulk format. The biosensor displayed a short DR of 0.1–0.6 mg/mL with a LOD of 0.062 mg/mL. A cross-reactivity study was performed in the presence of cytochrome c and bovine serum albumin (BSA) as interfering proteins and other parameters affecting the assay, like pH, were also tested. However, neither reproducibility between different batches nor repeatability were tested in this work.

**Figure 7:**
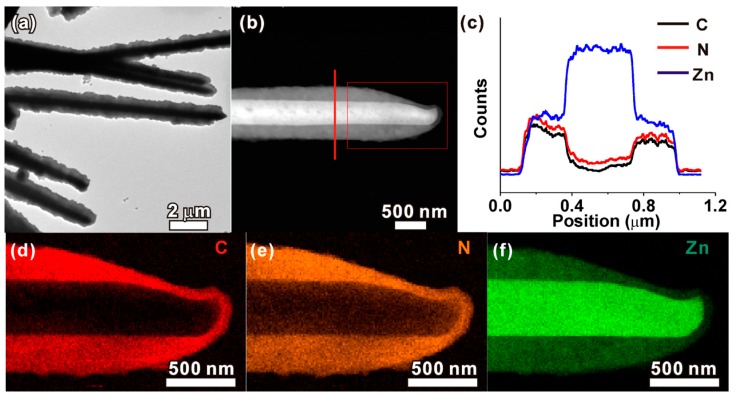
(**a**) Low-magnification TEM image of ZnO@ZIF-8 nanorods; (**b**) high-angle annular dark-field scanning transmission electron microscopy (HAADF-STEM) image of an individual ZnO@ZIF-8 nanorod; (**c**) cross-sectional compositional line profiles of ZnO/ZIF-8 recorded along the line marked in panel b; (**d**−**f**) elemental maps of C, N, and Zn concentrations in the ZnO@ZIF-8 nanorod recorded from the zone marked with a rectangle in panel b. Reprinted from reference [127], Copyright 2013, with permission from American Chemical Society.

Following a self-template strategy, Zhan et al. fabricated a photoelectrochemical biosensor for the detection of H_2_O_2_ in serum samples [127]. For that purpose, an ordered ZnO array was created electrochemically on the surface of a fluorinated tin oxide (FTO)-coated glass. The template was immersed in a solution containing 2MI as ligand in a mixture DMF:H_2_O, obtaining a ZnO@ZIF-8 nanotube array in a core–shell fashion after the thermal synthesis (Figure 7). Under light, ZnO generates holes and electrons and its combination with ZIF-8 allows the quantification of reductive species located within the MOF pores in terms of the current produced in the hybrid system. Although the LOD for the analyte was not specified, authors are able to perform the detection in a concentration range of 0–4 mM. No further studies on the reproducibility between batches or selectivity were tested in this work, that authors attribute to the molecule-size selective ability of the MOF.

An interesting localized surface plasmon resonance (LSPR)-based biosensor for the selective detection of glucose was prepared by Hang et al. following consecutive deposition of different materials [126]. The support material used as template consisted of a monolayer of colloid crystals of polystyrene (PS) nanospheres deposited onto glass substrates by self-assembling. The template was covered with a nanometric gold layer using magnetron sputtering deposition. Finally, the periodic Au nanosphere array was obtained after the thermal annealing of the material, calcining the polymer at 900 °C. The array was functionalized with PVP and finally immersed in a solution containing FeCl_3_ and BTC with DMF as solvent for the solvothermal synthesis of the polymer, resulting in a kind of core–shell structures attached to the glass support. In the last step, the hybrid chip was functionalized with 3-aminophenylboronic acid hemisulfate (PBA). Incubation of the chips in glucose solutions with different concentrations required 30 min to reach saturation and the variation of the initial signal was monitored with an UV-spectrometer. A short DR of 2–40 mM was obtained, and although other potential interferences did not produce critical changes in the optical signal, the level assayed for all of them was low, 4 mM. Further analytical details such as LOD, repeatability or stability were not evaluated, and the application for real samples was not demonstrated.

#### 4.3.2. Carbon-Based Cores

This last section deals with other possible materials used as cores not described before, those based not only on carbon derivatives such as graphene or nanotubes but also on organic polymers. Some of these examples are shown in Table 7. Due to their high conductivity, the vast majority of them are applied for the development of electrochemical biosensors.

A ratiometric electrochemical glucose biosensor based on a Cu-MOF was presented by Song et al. [130]. First, a three-dimensional macroporous carbon (3D-KSCs) was prepared and the MOF was created on the walls of the former material by mixing it with Cu(NO_3_)_2_ and BTC in a mixture of EtOH:H_2_O in a thermal synthesis. The new hybrid material was activated as an electrode and incubated with HAuCl_4_ to produce AuNPs after electrochemically treatment. In the last step, the electrode was incubated with glucose oxidase. The concentration of MOF onto the surface of 3D-KSCs seemed to be critical for the performance of the biosensor, finding a LOD of 14.77 μM and a linear range of 44.9 μM–19 mM, being applied for the quantification of glucose in serum samples with negligible cross-reactivity.

Another carbon-based material was selected to growth porphyrinic-based MOF crystals showing mimic peroxidase activity for the detection of H_2_O_2_ from cells [131]. First, nanoporous carbon with hexagonally ordered mesostructured was synthesized using SBA-15 silica as template and sucrose as carbon source. After calcination, the ordered mesoporous carbon (OMC) material was obtained. In parallel, [Fe_3_O(OOCCH_3_)_6_OH] crystals were produced by mixing Fe(NO_3_)_3_ and sodium acetate and recrystallized in DMF. Carbon-based material OMC was mixed with a solution containing these crystals, the iron(III)-based porphyrin TCPP as linker, TFA and DMF. After ultrasonic treatment, the solvothermal synthesis was performed and the hybrid material was obtained. It was mixed with Nafion and casted onto pre-treated GCE electrodes. The amperometric current response of the release flux of H_2_O_2_ was performed when incubating the electrodes in the presence of a suspension of cells. The growth of the MOF onto OMC resulted in less agglomerated polymers than those prepared in the absence of the carbon-based support, yielding more active sites exposed to the analyte. Additionally, OMC improved both the conductivity and stability of the final composite. A linear behavior was observed in the range 0.5–1830.5 μM with a LOD of 0.45 μM, excellent repeatability (< 5.5% RSD) and negligible cross-reactivity towards potential interferences, and with a moderate stability of, at least, two weeks.

An interesting example to create mesoporous MOF materials for the detection of H_2_O_2_ has been reported elsewhere [132]. This is an example of the use of other materials as templates, in this case PS nanobeads, to synthesize within its pores the MOF ZIF-8. Polymeric beads were swollen in methanol and mixed with the precursors Zn(NO_3_)_2_ and 2MI. After the synthesis at room temperature, the composite is centrifuged and calcined in a further step at 300 °C to eliminate the polymer matrix thus obtaining the MOF showing the complementary image of the swollen PS and introducing this artificial porosity. Finally, the MOF material was functionalized with cytochrome c. Then, after mixing the material with Nafion, it was deposited onto the surface of a screen-printed electrode for electrochemical detection using 3-ethylbenzothiazoline-6-sulfonic acid (ABTS) as electron mediator and showing higher activity than that obtained with the native cytochrome c. A DR of 0.09–3.6 mM was obtained, without selectivity towards other interfering species. No further discussion on the recoveries obtained for water, milk, and beer samples was given.

Another example to be highlighted consists on the fabrication of CuMOF-based nanocubes for the development of an electrochemical biosensor capable to detect lactate and glucose in sweat samples [133]. In the first step, graphene oxide paper (GOP) is fabricated from an aqueous suspension of GO sheets in a casting mold. After the evaporation of the solvent, GOP support material is obtained, further functionalized to include amino groups in the surface and finally electrochemically reduced to obtain graphene paper (GP). Fabrication of the MOF consists of an interfacial emulsion synthesis. The aqueous phase contained Cu(AcO)_2_ with PVP as surfactant. On the other hand, the oil phase was prepared dissolving BTC in 1-pentanol. Both solutions were mixed and stirred vigorously to form the emulsion, and the reaction started in the interface of the nano-droplets. Emulsion was broken by adding ethanol and the Cu-MOF nanocubes formed a close-packed layer in the interphase of the two phases. Amino-functionalized GP was dip-coated in this mixture and an ordered array of MOF nanocubes were obtained in the surface of the support material. The biosensor allowed the simultaneous detection of both lactate and glucose with DRs of 0.05–22.6 mM and 0.05–1775.5 μM and LODs of 5 μM and 30 nM, respectively. A broad variety of organic and inorganic interferences were tested demonstrating the high selectivity of the developed platform, showing a good fabrication reproducibility, with less than 2.61% RSD, and a good stability after 50 days.

**Figure 8:**
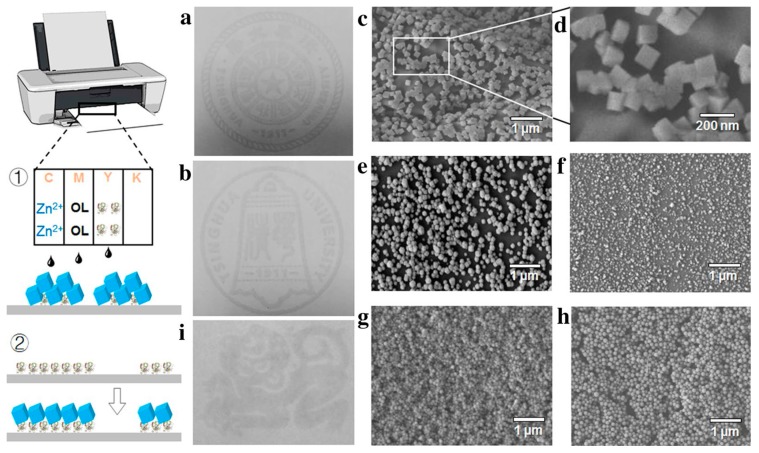
(**a**,**b**) Printed patterns formed by ZIF-8 crystals on filter paper, and patterns (approximately 5 × 5 cm) are badges of Tsinghua University. SEM images of pure ZIF-8 particles printed on: (**c**,**d**) filter paper (eight times printing); (**e**) PVC film (eight times printing); (**f**) PET film (eight times printing); (**g**) hydrophilic printing film (one time printing); (**h**) SEM image of ZIF-8 particles grown on PET film printed with protein; (**i**) a pattern (approximately 5 × 3 cm) of a monkey representing lunar year of 2016 formed by ZIF-8 particles grown on PET film printed with protein. Organic ligand 2MI is abbreviated as OL in the figure. Reprinted from reference [134], Copyright 2017, with permission from Springer Nature.

Hou and colleagues proposed a fluorescence imaging-based biosensor with a MOF printed onto the surface of a filter paper and other polymers following the ink-jet printing technique [134]. MOF components were loaded in the inks of the printer, i.e., Zn(NO_3_)_2_ and 2MI, with labeled cytochrome c (Figure 8). In the presence of H_2_O_2_, the biosensor resulted in a change of the fluorescence intensity yielding a LOD for this analyte of 20 mM and a DR of 20–120 mM. Although this work demonstrates for the first time the possibility of obtaining MOF materials for biosensor purposes using conventional printers, no further studies about the reproducibility between batches were tested and MOF-papers were not used for the analysis of the target molecule in real samples.

## 5. Conclusions and Future Perspectives

A new review based on MOFs for the development of biosensors has been presented. Works discussed herein have been recently published, most of them within the last two years. A classification depending on both the fabrication technique and the integration of the MOF in the sensor scheme has been followed. Those MOFs used as synthesized for biosensor purposes showed, in general, interesting optical properties that can be measured in batch-based assays depending on the concentration of the analyte. However, considering the same materials deposited onto different substrates in a “grafting to” approach resulted in the development of electrochemical biosensors. Most of the works published to date belongs to this group of MOF-based biosensors. In order to improve the conductivity of bulk materials some strategies have been followed: restricted growth of the material in 2D to produce nanosheets; impregnation and further reduction with metal salts to create metal nanoparticles within the polymeric matrix; or calcination of the material itself prior to its application. On the other hand, alternative synthetic routes based on the “grafting from” approach have drawn attention to core–shell materials and their enhanced properties over the individual counterparts. In any case, a singular behavior has been observed: those biosensors where the core material is a metal or metal nanoparticles are mainly used for optical transduction, while if the substrate is a carbon-based material, the biosensor is used for electrochemical applications.

As MOF-based materials fabricated under these conditions are still under development, and new micro- and nanofabrication procedures are being adapted for their synthesis, literature is not extensive enough to stablish a deeper distribution. However, taking into account the interest of the hybrid materials, one could expect that in the nearly future this classification may be widened. Taking other polymer counterparts as references, such as MIPs, one could expect that in the future clearly distributions based on the nature of the core materials would be followed. The opinion of this author is that “grafting from” approaches will be exploited considerably as the integration of MOF materials with either recognition or transducer elements results in more reproducible, robust and reliable hybrids capable to fulfill the future sensing requirements. The use of magnetic nanoparticles as cores would decrease time analyses and ease the measurements in batch-based assays, while using luminescent scaffolds such as QDs or UCNPs would result in the development of more accurate and sensitive optical devices. On the other hand, those carbon-based MOF hybrid materials would considerably increase the amount of electrochemical sensing schemes due to their unique conductive properties and the perfect integration with the polymer. Due to the broad amount of possibilities choosing these carbon-based materials—including GO, GOP, GP, KSC or even conductive polymers, among others—it is expected that the fabrication of electrochemical sensing devices would considerably surpass other transduction schemes.

## Figures and Tables

**Table 1:** Biosensors using raw MOFs.

Composition (Metal Precursor/Organic Ligand/Solvent/Modulator)	Sensing	Analyte	DR	LOD	Sample	Ref.
Zn(NO_3_)_2_/H_4_TCPB/DMF	FL	Parathion-methyl	1 μg/kg–10 mg/kg	0.12 μg/kg	Water	[59]
Fe/BTC/H_2_O/HF,HNO_3_	FL	Choline	0.5–10 μM	0.027 μM	Milk	[60]
		Acetylcholine	0.1–10 μM	0.036 μM	Serum	
CuSO_4_/bpe,H_3_DcbcpBr/H_2_O	FL	Dengue virus	1–60 nM	332 ppm		[61]
		Zika virus	0.5–70 nM	192 ppm		
Cu(NO_3_)_2_/H_2_dcbbBr/H_2_O	FL	Gastric cancer miRNAs	*	91–559 pM		[62]
PbCl_2_,CdCl_2_/2ATPA/DMF:EtOH	EC	CEA	0.3–3 ng/mL	0.03 pg/mL	Serum	[63]
		AFP		0.1 pg/mL		
FeCl_3_/2ATPA/H_2_O	PL	*S. aureus*	40–41 × 0^8^ CFU/mL	31 CFU/mL	Cream pastry	[64]
FeCl_3_/2ATPA/DMF/AcOH	CL	H_2_O_2_	0.1–10 μM	0.025 μM	Milk	[65]
FeCl_3_/2ATPA/H_2_O/AcOH,Pluronic F127	FL	BPA	5 × 10^−14^–2 × 10^−9^ M	4.1 × 10^−14^ M		[66]
Cd(NO_3_)_2_/2ATPA(Na)/H_2_O	FL	Parathion	1 ppb–1 ppm	1 ppb	Serum	[67]
Zn(NO_3_)_2_/Cbdcp,bpe/DMF or DMF:H_2_O/Aspirin/5FU	FL	HIV dsDNA	1–80 nM	10 pM		[68]
HfCl_4_/AQPDC,DBP(Pt)/DMF/AcOH	PL/FL	O_2_	8–81 mmHg	n.d.	Cells	[69]
	Imaging					
ZrCl_4_/2ATPA/DMF/AcOH	FL	Hg^2+^	0.1–10 μM	17.6 nM	Water	[70]
Dy(NO_3_)_3_/H_3_DcbcpBr/H_2_O/NaOH	FL	Ebola virus	5–50 nM	160 pM		[71]
Cu(NO_3_)_2_/H_2_dcbbBr/H_2_O	FL	HIV dsDNA	1–120 nM	1.42 nM		[72]
Zn(NO_3_)_2_/2MI/H_2_O	UV–vis	H_2_O_2_	0–800 μM	1.0 μM	Sewage	[73]
		Phenol	0–200 μM			
CuSO_4_/DTOA/H_2_O	FL	HIV virus	10–100 nM	3 nM		[74]
		Thrombin	5–100 nM	1.3 nM		
CuSO_4_/H_3_CmdrpBr,dps/H_2_O/NaOH	FL	HIV dsDNA	10–50 nM	196 pM		[75]
		Sudan RNA		73 pM		
Cr(NO_3_)_3_/TPA/H_2_O/HF	FL	DNA	0.1–14 nM	73 pM		[76]
Gd(NO_3_)_3_/TIA/DMF:H_2_O	FL	DNA	0–50 nM	n.d.		[77]
Eu(NO_3_)_3_/TDA/EtOH	FL	H_2_O_2_	5–150 μM	n.d.	Plasma	[78]
Cu(NO_3_)_2_/BTC/DMF:H_2_O:EtOH	CL	Dopamine	0.01–0.70 μM	2.3 nM	Urine	[79]
					Plasma	
Al(NO_3_)_3_/B4C/H_2_O	FL	HA	0.05–8 mg/mL	9 μg/mL	Urine	[80]
Eu(NO_3_)_3_,Tb(NO_3_)_3_/H_2_bpdc/MeOH:CHCl_3_	FL	LPA	1.4–43.3 μM	n.d.		[81]
Zn(AcO)_2_/BPDC,adenine/DMF:H_2_O/HNO_3_	FL	DCA	50 nM–1 μM	34 nM	Serum	[82]

* Five different analytes with their corresponding DRs were analyzed in this work; LOD value corresponds herein to the interval for all of them; n.d.: not determined; H_4_TCPB: 1,2,4,5-tetrakis(4-carboxyphenyl) benzene; BTC: trimesic acid; bpe: 1,2-bis(4-pyridyl)ethylene; H_3_DcbcpBr: *N*-(3,5-dicarboxylbenzyl)-(3-carboxyl) pyridinium bromide; H_2_dcbbBr: 1-(3,5-dicarboxybenzyl)-4,4′-bipyridinium bromide; 2ATPA: 2-aminoterephtalic acid; Cbdcp: *N*-(4-carboxybenzyl)-(3,5-dicarboxyl)pyridinium; 5FU: 5-fluorouracil; AQPDC: amino-quaterphenyldicarboxylic acid; DBP(Pt): Pt-5,15-di(*p*-benzoato)porphyrin; DTOA: dithiooxamide; H_3_CmdrpBr: *N*-carboxymethyl-3,5-dicarboxylpyridinium bromide; dps: 4,4′-dipyridyl sulfide; TPA: terephtalic acid; TIA: 5-triazoleisophtalic acid; TDA: 2,2′-thiodiacetic acid; B4C: 1,2,4,5-benzenetetracarboxylic acid; H_2_bpdc: 2,2′-bipyridine-6,6′-dicarboxylic acid; BPDC: 4,4′-biphenyldicarboxylate; miRNA: microRNA; CEA: carcinoembryonic antigen; AFP: alpha-fetoprotein; BPA: bisphenol A; HIV: human immunodeficiency virus; dsDNA: double-stranded DNA; HA: hippuric acid; LPA: lysophosphatidic acid; DCA: dipicolinic acid; FL: fluorescence; EC: electrochemical; PL: photoluminescence; CL: chemiluminescence; UV–vis: ultraviolet–visible.

**Table 2:** Biosensors using bulk MOFs grafted to different substrates.

Composition (Metal Precursor/Organic Ligand/Solvent/Modulator)	Sensing	Analyte	DR	LOD	Sample	Ref.
CrCl_3_/TPA/H_2_O	EC	H_2_O_2_	25–500 μM	3.52 μM	Serum	[83]
FeCl_3_/2ATPA/DMF/AcOH	EC	miRNA-122	0.01 fM–10 pM	0.003 fM	Serum	[84]
					Blood	
FeCl_3_/2ATPA/DMF/AcOH	EC	Pb^2+^	0.005–1000 nM	2 pM	Water	[85]
Zn(NO_3_)_2_/BPDC,adenine, Ru(bpy)_3_Cl_2_/DMF:H_2_O/HNO_3_	ECL	miRNA-155	0.8 fM–1 nM	0.3 fM	Serum	[86]
ZrCl_4_/TCPP/DMF/BA	FL	p53 gene	0.01–10 nM	0.005 nM	Serum	[87]
		PSA	0.05–10 ng/mL	0.01 ng/mL		
ZrCl_4_/TPA/DMF/AcOH	PEC	PKA	0.005–0.065 U/mL	0.0049 U/mL	Cells	[88]
CrO_3_/BTC,BBDC/H_2_O/HF	EC	H_2_O_2_	0.5–3000 μM	0.1 μM	Cells	[89]
FeCl_3_/2ATPA/DMF/AcOH	ECL	MUC1	1 fg/mL–1 ng/mL	0.26 fg/mL	Cells	[90]
CeCl_3_/2ATPA/H_2_O:2-propanol	EC	ATP	10 nM–1000 μM	5.6 nM	Serum	[91]
Cr(NO_3_)_3_/TPA/H_2_O/HF	FL	Thrombin	50 pM–100 nM	15 pM	Serum	[92]
		OTC	10 nM–2 μM	4.2 nM	Duck	
FeCl_3_/H_3_TAB/DMF/TFA	EC	H_2_O_2_	0.5 μM–5 mM	0.09 μM		[93]
H_3_PMo_12_O_40_,CuCl_2_/1,10-phen/H_2_O	EC	Dopamine	10^−6^–2 × 10^−4^ M	80.4 × 10^−9^ M	Serum	[94]
Fe(AcO)_3_/FA/MeOH:H_2_O/NaOH	UV–vis	H_2_O_2_	2 × 10^−6^–2.03 × 10^−5^ M	5.62 × 10^−7^ M		[95]
		AA	2.57 × 10^−6^–1.01 × 10^−5^ M	1.03 × 10^−6^ M		
FeCl_3_/TPA/DMF	EC	H_2_O_2_	0.1–2000 μM	0.075 μM	Water	[96]
		NO_2_^−^	0.4–7000 μM	0.36 μM		
ZrCl_4_/2ATPA/DMF/AcOH	EC	KANA	0.002–100 nM	0.16 pM	Milk	[97]
		CAP		0.19 pM		
Cu(NO_3_)_2_/beb,H_2_ada/H_2_O	EC	H_2_O_2_	0.05–3 μM	0.014 μM		[98]
FeCl_3_/TCPP/DMF:EtOH/HCl	EC	Pb^2+^	0.03–1000 nM	0.02 nM	Water	[99]
					Juices	
					Serum	
Cu(NO_3_)_2_/TCPP,4bpy/Acetone:H_2_O/NaOH	EC	NO_2_^−^	3.5–2800 μM	1.1 μM	Pickle	[100]
					Juice	
CuCl_2_/H_2_Leu/CH_3_CN:EtOH/LiOH	EC	MBZ	0.001–0.1 mM	1.3 μM		[101]

TPA: terephtalic acid; 2ATPA: 2-aminoterephtalic acid; BPDC: 4,4′-biphenyldicarboxylate; Ru(bpy)_3_Cl_2_: tris(bipyridine) ruthenium (II); TCPP: tetrakis (4-carboxyphenyl) porphyrin; BTC: trimesic acid; BBDC: 5-boronobenzene-1,3-dicarboxylic acid; H_3_TAB: 4,4′,4′′-*s*-triazine-2,4,6-triyl-tribenzoic acid; 1,10-phen: 1,10-phenantroline; FA: fumaric acid; miRNA: microRNA; PSA: prostate specific antigen; PKA: protein kinase A; MUC1: mucin 1; ATP: adenosine triphosphate; OTC: oxytetracycline; AA: ascorbic acid; KANA: kanamycin; CAP: chloramphenicol; MBZ: α-methylbenzylamine; EC: electrochemical; ECL: electrochemiluminescence; FL: fluorescence; PEC: photoelectrochemical; UV–vis: ultraviolet–visible.

**Table 3:** Biosensors using MOF nanosheets grafted to different substrates.

Composition (Metal Precursor/Organic Ligand/Solvent/Modulator)	Sensing	Analyte	DR	LOD	Sample	Ref.
Zn(NO_3_)_2_/[Ru(dcbpy)_3_]^2+^/PrOH:H_2_O	ECL	miRNA-141	1 fM–10 pM	0.3 fM	Serum	[102]
Cu(NO_3_)_2_/TCPP/DMF:EtOH/TFA,PVP	FRET	CAP	0.001–10 ng/mL	0.3 pg/mL	Milk	[103]
					Fish	
Cu(NO_3_)_2_/2ATPA/DMF:CH_3_CN	FL	HXA	10–2000 μM	3.93 μM	Fish	[104]
Ni(NO_3_)_2_/TPA/DMF:H_2_O/NaOH	EC	Glucose	4–5664 μM	0.8 μM	Serum	[105]
ZrOCl_2_/H_3_NBB/DEF/TFA	EC	MUC1	0.001–0.5 ng/mL	0.12 pg/mL	Serum	[106]
	SPR			0.65 pg/mL		

[Ru(dcbpy)_3_]^2+^: tris(4,4′-dicarboxylicacid-2,2′-bipyridyl) ruthenium(II); PrOH: propanol; TCPP: tetrakis (4-carboxyphenyl) porphyrin; TFA: trifluoroacetic acid; PVP: polyvinylpyrrolidone; 2ATPA: 2-aminoterephtalic acid; TPA: terephtalic acid; H_3_NBB: 4′,4′″,4′″″-nitrilotris([1,1′-biphenyl]-4-carboxylic acid); DEF: *N*,*N*-diethylformamide; miRNA: microRNA; CAP: chloramphenicol; HXA: hypoxanthine; MUC1: mucin 1; ECL: electrochemiluminescence; FRET: Förster resonance energy transfer; FL: fluorescence; EC: electrochemical; SPR: surface plasmon resonance.

**Table 4:** Biosensors using metal NP@MOFs grafted to different substrates.

Composition (Metal Precursor/Organic Ligand/Solvent/Modulator/NP)	Sensing	Analyte	DR	LOD	Sample	Ref.
Co(NO_3_)_2_/2MI/MeOH:EtOH/Ag	EC	Glucose	2–1000 μM	0.66 μM		[107]
FeCl_3_/2ATPA/DMF/AcOH/Au	EC	Gal-3	100 fg/mL–50 ng/mL	33.33 fg/mL	Serum	[108]
ZrCl_4_/TPA/DMF/TFA,HCl/Ag	EC	CEA	0.01–10 ng/mL	0.31 pM	Serum	[109]
	SPR		4.0–250 ng/mL	0.3 ng/mL		
Co(NO_3_)_2_/2MI/H_2_O/CTAB/AuPt	EC	LAG-3	0.01 ng/mL–1 μg/mL	1.1 pg/mL	Serum	[110]
FeCl_3_/2ATPA/DMF/AcOH/AuPt	EC	ADRB1	1 fM–10 nM	0.21 fM	Serum	[111]
FeCl_3_/2ATPA/DMF/AcOH/Pt	EC	FGFR3	0.1 fM–1 nM	0.033 fM	Serum	[112]
PbCl_2_/β-CD/H_2_O:cyclohexanol/Et_3_N/Au	ECL	Insulin	0.1 pg/mL–10 ng/mL	0.042 pg/mL		[113]
Cu(NO_3_)_2_/2ATPA/DMF:EtOH/PVP/Au	EC	miRNA-155	1 fM–10 nM	0.35 fM	Serum	[114]

2MI: 2-methylimidazole; 2ATPA: 2-aminoterephtalic acid; TPA: terephtalic acid; TFA: trifluoroacetic acid; CTAB: cetyltrimethyl ammonium bromide; β-CD: β-cyclodextrin; PVP: polyvinylpyrrolidone; Gal-3: galectin-3; CEA: carcinoembryonic antigen; LAG-3: lymphocyte activation gene-3 protein; ADRB1: adrenergic receptor gene; FGFR3: fibroblast growth factor receptor 3; miRNA: microRNA; EC: electrochemical; SPR: surface plasmon resonance; ECL: electrochemiluminescence.

**Table 5:** Biosensors using calcined MOFs grafted to different substrates.

Composition (Metal Precursor/Organic Ligand/Solvent/Modulator)	Sensing	Analyte	DR	LOD	Sample	Ref.
NiCl_2_/TPA/DMF	EC	Glucose	0.4–900 μM	0.1 μM	Serum	[115]
Fe(NO_3_)_3_,ZrCl_4_, La(NO_3_)_3_/2ATPA/DMF	EC	Parathion-methyl	10^−12^–10^−8^	3.2 × 10^−13^		[116]
			5 × 10^−13^–5 × 10^−9^	1.8 × 10^−13^		
			10^−13^–5 × 10^−9^ g/mL	5.8 × 10^−14^ g/mL		
CoCl_2_/2MI/MeOH	EC	Glucose	5 × 10^−12^–2.05 × 10^−10^ M	2 × 10^−12^ M	Serum	[117]
Zn(NO_3_)_2_/2MI/MeOH	PEC	Alkaline phosphatase	2–1500 U/L	1.7 U/L	Serum	[118]
Al(NO_3_)_3_/NDC/H_2_O	EC	Glucose	0.07–0.99 mM	0.065 mM		[119]
Fe(NO_3_)_3_/FA/DMF	FL	DNA	3–150 nM	1 nM	Serum	[120]

TPA: terephtalic acid; 2ATPA: 2-aminoterephtalic acid; 2MI: 2-methylimidazole; NDC: 1,4-naphtalenedicarboxylic acid; FA: fumaric acid; EC: electrochemical; PEC: photoelectrochemical; FL: fluorescence.

**Table 6:** Biosensors using MOFs grafted from metal/metal oxides substrates.

Composition (Metal Precursor/Organic Ligand/Solvent/Modulator/Core)	Sensing	Analyte	DR	LOD	Sample	Ref.
FeCl_3_/TPA/DMF/NaOH/Fe_3_O_4_	UV–vis	Glutathione	0.55–3 μM	36.9 nM	Serum	[121]
Cu(NO_3_)_2_/BTC/EtOH:H_2_O/Fe_3_O_4_,g-C_3_N_4_	FL	OTA	5–160 ng/mL	2.57 ng/mL	Corn	[122]
Zn(NO_3_)_2_/2MI/EtOH:H_2_O/GO-CaCO_3_@PDA	EC	Glucose	1 μM–3.6 mM	0.333 μM	Serum	[123]
Zn(NO_3_)_2_/2MI/H_2_O/CdTe-QDs@PVP	FL	H_2_O_2_	1–100 nM	0.29 nM	Serum	[124]
		Urate oxidase	0.1–50 U/L	0.024 U/L		
		Glucose oxidase	1–100 U/L	0.26 U/L		
Cu(NO_3_)_2_/BTC/DMF:EtOH/Y-Yb-Er-UCNPs@PAA	FL	BHB	0.1–0.6 mg/mL	0.062 mg/mL		[125]
FeCl_3_/BTC/DMF/PS@Au@PVP	LSPR	Glucose	2–40 mM	n.d.		[126]
2MI/DMF:H_2_O/Glass@FTO@ZnO	PEC	H_2_O_2_	0–4 mM	n.d.	Serum	[127]
Zn(AcO)_2_/2MI/H_2_O/AuNRs	LSPR	HSA	250–1000 ng/mL	130 ng/mL		[128]
TiTB/2ATPA/DMF:EtOH/TiO_2_	PEC	Acetochlor	0.02–200 nM	0.003 nM	Strawberry	[129]
					Tomato	
					Cucumber	
					Greens	

n.d.: not determined; TPA: terephtalic acid; BTC: trimesic acid; g-C_3_N_4_: graphitic carbon nitride; 2MI: 2-methylimidazole; GO: graphene oxide; PDA: polydopamine; QD: quantum dot; PVP: polyvinylpyrrolidone; UCNP: up-conversion nanoparticle; PAA: polyacrylic acid; PS: polystyrene; FTO: fluorinated tin oxide; AuNRs: gold nanorods; TiTB: tetrabutyl titanate; 2ATPA: 2-aminoterephtalic acid; OTA: ochratoxin A; BHB: bovine hemoglobin; HAS: human serum albumin; UV–vis: ultraviolet–visible; FL: fluorescence; EC: electrochemical; LSPR: localized surface plasmon resonance; PEC: photoelectrochemical.

**Table 7:** Biosensors using MOFs grafted from carbon-based substrates.

Composition (Metal Precursor/Organic Ligand/Solvent/Modulator/Core)	Sensing	Analyte	DR	LOD	Sample	Ref.
Cu(NO_3_)_2_/BTC/EtOH:H_2_O/3D-KSCs	EC	Glucose	44.9 μM–19 mM	14.77 μM	Serum	[130]
[Fe_3_O(OOCCH_3_)_6_OH]/TCPP(Fe)/DMF/TFA/OMC	EC	H_2_O_2_	0.5–1830.5 μM	0.45 μM	Cells	[131]
Zn(NO_3_)_2_/2MI/MeOH/PS	EC	H_2_O_2_	0.09–3.6 mM	n.d.	Water	[132]
					Milk	
					Beer	
Cu(AcO)_2_/BTC/H_2_O:1-pentanol/PVP/GP	EC	Lactate	0.05–22.6 mM	5 μM	Sweat	[133]
		Glucose	0.05–1775.5 μM	30 nM		
Zn(NO_3_)_2_/2MI/ink/paper	FL	H_2_O_2_	20–120 mM	20 mM		[134]
AlCl_3_/H_3_TAB/DMF/TFA/3D-KSCs	EC	H_2_O_2_	0.387 μM–1.725 mM	0.127 μM		[135]
Cu(NO_3_)_2_/BTC/EtOH:H_2_O/GO	EC	H_2_O_2_	1 μM–5.6mM	0.049 μM	Serum	[136]
Tb(NO_3_)_3_/H_3_TAB/MeOH,H_2_O,DMA/3D-KSCs	EC	H_2_O_2_	3.02–640 μM	0.996 μM	Disinfector	[137]
ZrOCl_2_/TCPP/DMF/BA/PEDOT NTs	EC	Dopamine	2 × 10^−6^–270 × 10^−6^ M	4 × 10^−8^ M	Cells	[138]
Cu(OH)_2_/BTC/EtOH:H_2_O/GCE	EC	Glucose	2 μM–4 mM	0.6 μM	Serum	[139]
Zn(NO_3_)_2_/2MI/MeOH/PVP/GO	EC	H_2_O_2_	0.02–6 mM	3.4 μM		[140]

n.d.: not determined; BTC: trimesic acid; KSC: macroporous carbon; TCPP: tetrakis (4-carboxyphenyl) porphyrin; TFA: trifluoroacetic acid; OMC: ordered mesoporous carbon; 2MI: 2-methylimidazole; PS: polystyrene; PVP: polyvinylpyrrolidone; GP: graphene paper; H_3_TAB: 4,4′,4′′-*s*-triazine-2,4,6-triyl-tribenzoic acid; DMA: *N*,*N*-dimethylacetamide; BA: benzoic acid; PEDOT NTs: poly(3,4-ethylenedioxythiophene) nanotubes; GCE: glassy carbon electrode; GO: graphene oxide; EC: electrochemical.

## Abbreviations

1,10-phen	1,10-phenantroline
2ATPA	2-aminoterephtalic acid
2MI	2-methylimidazole
4bpy	4,4′-bipyridine
5FU	5-fluorouracil
AA	ascorbic acid
Ab	antibody
ABTS	3-ethylbenzothiazoline-6-sulfonic acid
AcO	acetate
AcOH	acetic acid
ADRB1	adrenergic receptor gene
AFP	alpha-fetoprotein
AgNPs	silver nanoparticles
APTES	(3-aminopropyl)-triethoxysilane
AQPDC	amino-quaterphenyldicarboxylic acid
ATP	adenosine triphosphate
AuNPs	gold nanoparticles
AuNRs	gold nanorods
AuPtNPs	gold and platinum nanoparticles
AUR	Amplex UltraRed
B4C	1,2,4,5-benzenetetracarboxylic acid
BA	benzoic acid
BBDC	5-boronobenzene-1,3-dicarboxylic acid
β-CD	β-cyclodextrin
beb	1,4-bis(2-ethylbenzimidazol-1-ylmethyl) benzene
BHB	bovine hemoglobin
BTC	trimesic acid
BPA	bisphenol A
BPDC	4,4′-biphenyldicarboxylate
bpe	1,2-bis(4-pyridyl)ethylene
bpea	1,2-bis(4-pyridyl)ethane
BSA	bovine serum albumin
BTC	trimesic acid
CAP	chloramphenicol
Cbdcp	*N*-(4-carboxybenzyl)-(3,5-dicarboxyl)pyridinium
cDNA	capture DNA
CEA	carcinoembryonic antigen
CL	chemiluminescence
CTAB	cetyltrimethyl ammonium bromide
CPE	carbon paste electrode
DBP(Pt)	Pt-5,15-di(*p*-benzoato)porphyrin
DCA	dipicolinic acid
DEF	*N*,*N*-diethylformamide
DMA	*N*,*N*-dimethylacetamide
DMF	*N*,*N*-dimethylformamide
DNA	deoxyribonucleic acid
dps	4,4’-dipyridyl sulfide
DPV	differential pulse voltammetry
DR	dynamic range
dsDNA	double-stranded DNA
DTOA	dithiooxamide
EC	electrochemical
ECL	electrochemiluminescence
EIS	electrochemical impedance spectroscopy
Et_3_N	triethylamine
EtOH	ethanol
FA	fumaric acid
FAM	carboxyfluorescein
FGFR3	fibroblast growth factor receptor 3
FL	fluorescence
FRET	Förster resonance energy transfer
FTO	fluorinated tin oxide
Gal-3	galectin-3
g-C_3_N_4_	graphitic carbon nitride
GCE	glassy carbon electrode
GO	graphene oxide
GOP	graphene oxide paper
GP	graphene paper
H_2_ada	1,3-adamantanediacetic acid
H_2_bpdc	2,2′-bipyridine-6,6′-dicarboxylic acid
H_2_dcbbBr	1-(3,5-dicarboxybenzyl)-4,4’-bipyridinium bromide
H_2_Leu	*N*-(2-hydroxybenzyl)-L-leucine
H_3_CmdrpBr	*N*-carboxymethyl-3,5-dicarboxylpyridinium bromide
H_3_DcbcpBr	*N*-(3,5-dicarboxylbenzyl)-(3-carboxyl) pyridinium bromide
H_3_NBB	4′,4′″,4′″″-nitrilotris([1,1′-biphenyl]-4-carboxylic acid)
H_3_TAB	4,4′,4′′-*s*-triazine-2,4,6-triyl-tribenzoic acid
H_4_TCPB	1,2,4,5-tetrakis(4-carboxyphenyl) benzene
HA	hippuric acid
HIV	human immunodeficiency virus
HKUST	Hong Kong University of Science and Technology
HSA	human serum albumin
HXA	hypoxanthine
ITO	indium tin oxide
KANA	kanamycin
KSC	macroporous carbon
LAG-3	lymphocyte activation gene-3 protein
LOD	limit of detection
LPA	lysophosphatidic acid
LSPR	localized surface plasmon resonance
MBA	*N*,*N*-methylenebisacrylamide
MBZ	α-methylbenzylamine
MeOH	methanol
MIL	materials of Institute Lavoisier
MIP	molecularly imprinted polymer
miRNA	microRNA
MOF	metal organic framework
MUC1	mucin 1
N-G	nitrogen-doped graphene sheets
N-GNRs	nitrogen-doped graphene nanoribbons
NIPAAM	*N*-isopropyl acrylamide
NDC	1,4-naphtalenedicarboxylic acid
NPC	nanoporous carbon
OMC	ordered mesoporous carbon
OTA	ochratoxin A
OTC	oxytetracycline
PAA	polyacrylic acid
PBA	3-aminophenylboronic acid hemisulfate
PCN	porous coordination network
PDA	polydopamine
PdNPs	palladium nanoparticles
PEC	photoelectrochemical
PEDOT NTs	poly(3,4-ethylenedioxythiophene) nanotubes
PKA	protein kinase A
PL	photoluminescence
PrOH	propanol
PS	polystyrene
PSA	prostate specific antigen
PVP	polyvinylpyrrolidone
QD	quantum dot
rGO	reduced graphene oxide
RNA	ribonucleic acid
ROX	5(6)-carboxyrhodamine, triethylammonium salt
RSD	relative standard deviation
[Ru(bpy)_3_]^2+^	tris(bipyridine) ruthenium (II)
[Ru(dcbpy)_3_]^2+^	tris(4,4′-dicarboxylicacid-2,2′-bipyridyl) ruthenium(II)
sDNA	signal DNA
SEM	scanning electron microscopy
SPR	surface plasmon resonance
ssDNA	single-stranded DNA
TCPP	tetrakis (4-carboxyphenyl) porphyrin
TDA	2,2′-thiodiacetic acid
TEM	transmission electron microscopy
TFA	trifluoroacetic acid
TIA	5-triazoleisophtalic acid
TiTB	tetrabutyl titanate
TMB	3,3′,5,5′-tetramethylbenzidine
TPA	terephtalic acid
UCNPs	up-conversion nanoparticles
UiO	Universitetet i Oslo
UV–vis	ultraviolet–visible
ZIF	zeolitic imidazolate framework
